# Lamotrigine-Induced Toxic Epidermal Necrolysis with Multiorgan Failure, Pneumatosis Intestinalis and Chronic Ocular Complications

**DOI:** 10.3390/life16081238

**Published:** 2026-07-27

**Authors:** Jakub Szrama, Piotr Smuszkiewicz, Amadeusz Woźniak, Ashish Lohani, Krzysztof Zwoliński, Paweł Sobczyński, Nina Łabędź, Aleksandra Dańczak-Pazdrowska, Paweł Pazdrowski, Anna Mikołajczyk-Lorkiewicz, Joanna Wojciechowska, Marcin Stopa, Adriana Polańska

**Affiliations:** 1 Department of Anesthesiology, Intensive Therapy and Pain Management, Poznan University of Medical Sciences, 60-355 Poznan, Poland; piotr.smuszkiewicz@usk.poznan.pl (P.S.); amadeusz.wozniak@usk.poznan.pl (A.W.); ashish.lohani@usk.poznan.pl (A.L.); krzysztof.zwolinski@usk.poznan.pl (K.Z.); pawel.sobczynski@usk.poznan.pl (P.S.); 2 Department of Dermatology, Poznan University of Medical Science, 60-355 Poznan, Poland; nina.labedz@usk.poznan.pl (N.Ł.); aleksandra.danczak-pazdrowska@usk.poznan.pl (A.D.-P.); 3 Department of Ophthalmology, Chair of Ophthalmology and Optometry, Poznan University of Medical Sciences, 60-780 Poznan, Poland; pawel.pazdrowski@usk.poznan.pl (P.P.); anna.mikolajczyk-lorkiewicz@usk.poznan.pl (A.M.-L.); joanna.wojciechowska@usk.poznan.pl (J.W.); marcin.stopa@usk.poznan.pl (M.S.); 4 Department of Dermatology and Venerology, Poznan University of Medical Sciences, 60-780 Poznan, Poland; adriana.polanska@usk.poznan.pl

**Keywords:** lamotrigine, toxic epidermal necrolysis, multiorgan failure, intensive therapy, pneumatosis intestinalis, portal venous gas, chronic ocular complications

## Abstract

Toxic epidermal necrolysis (TEN) is a rare, life-threatening mucocutaneous adverse drug reaction associated with extensive epidermal necrosis and severe systemic complications. We report the case of a 25-year-old woman who developed severe lamotrigine-induced TEN four weeks after treatment initiation. The disease rapidly progressed to involve approximately 60% of the body surface area, requiring intensive care unit admission, mechanical ventilation, and multidisciplinary management. Treatment included high-dose intravenous methylprednisolone, intravenous immunoglobulins, etanercept, plasmapheresis, and comprehensive supportive care. During hospitalization, the patient developed gastrointestinal complications manifested by pneumatosis intestinalis and portal venous gas, raising suspicion of bowel ischemia and prompting exploratory laparotomy. Following treatment, gradual clinical improvement and complete resolution of the cutaneous lesions were achieved. However, persistent ocular sequelae developed, including meibomian gland dysfunction, punctate epithelial erosions, conjunctival scarring, trichiasis, tear film instability, and early corneal neovascularization, requiring long-term ophthalmological follow-up. To provide clinical context, a narrative review of the literature on the diagnosis, multidisciplinary management, gastrointestinal manifestations, ocular complications, and systemic treatment of TEN was performed. A comprehensive literature search was conducted to identify relevant articles focusing on the intensive care management and specific organ-related manifestations of Toxic Epidermal Necrolysis (TEN). The gathered literature was narratively synthesized to present a cohesive update on current clinical practices and management controversies. This case highlights the potentially devastating multiorgan course of TEN and emphasizes the importance of early recognition, specialized intensive care, and long-term multidisciplinary follow-up.

## 1. Introduction

Stevens-Johnson syndrome (SJS) and toxic epidermal necrolysis (TEN) are serious, often life-threatening mucocutaneous diseases caused by a pathological immune reaction most frequently induced by drugs or their metabolites [1]. The incidence of SJS/TEN ranges from two to thirteen cases per million per year, affecting all age groups, with more frequent presentation in women [1,2,3,4]. There is a genetic predisposition to the disease related to some leukocyte antigen (HLA) types, with HLA-B*1502 associated with almost an 80-fold increased risk (OR 79.8; 95% CI 28.45–224.06) of carbamazepine-induced SJS/TEN in the East Asian population [5], while HLA-B*5801 is associated with SJS/TEN due to allopurinol exposure in European and Asian populations [6]. Patients with hematologic malignancies [7], autoimmune diseases like lupus erythematosus [8], or those with HIV infection [9] also pose an increased risk of developing SJS/TEN. The mortality rate for SJS is around 5.4–10%; however, TEN, being a more severe form, has a mortality rate of around 15–50%, reaching the highest values in patients with older age and comorbidities [2,3,4,7,10,11,12].

According to results of a retrospective study from the United States, almost 90% of SJS/TEN cases are drug-induced, with antimicrobials (trimethoprim/sulfamethoxazole, β-lactam antibiotics, fluoroquinolones), antiepileptics (including lamotrigine, phenytoin, and carbamazepine), allopurinol, and nonsteroidal anti-inflammatory medications being responsible for most of the cases [10]. In most cases, the culprit drugs were initiated 1–3 weeks before the onset of the symptoms, with an unlikely diagnosis of SJS/TEN after 8 weeks of drug administration [10]. Following the mentioned analysis of 377 cases in the United States, in around 3% of cases, there was a preceding infection, and in almost 7% of cases, the cause was unknown [10].

SJS/TEN typically begins with flu-like symptoms lasting 1–3 days, followed by skin tenderness, photophobia, conjunctival itching, and dysphagia [11]. Typical initial skin lesions are known as dusky atypical targets. The central component of these lesions is an erythematous macule or papule, which is dull or dusky in color. Individual targetoid papules and larger coalescing areas of dusky erythema evolve into vesicles or bullae over 1 to 3 days or widespread denuded skin [13].

The pathogenesis of SJS/TEN includes keratinocyte apoptosis induced by natural killer cells and CD8 lymphocytes, which release cytotoxic mediators, tumor necrosis factor, perforin/granzyme, and granulysin [14,15]. Granulysin, which is produced and secreted by both the NK and CD8 cells, is highly expressed in the blister fluid, correlating with the severity of SJS/TEN. It is able to induce a cytotoxic effect on the keratinocytes in a mouse model, and is also a potential therapeutic target for future research [16].

SJS/TEN are characterized by widespread keratinocyte death resulting in full-thickness denudation of the skin and mucosa, leaving the patients highly susceptible to a life-threatening infection [11]. SJS and TEN are distinguished by the body surface area (BSA) affected by the lesions, with less than < 10% of BSA termed SJS, more than > 30% BSA termed TEN, while the intermediate involvement is termed SJS/TEN overlap [1,13].

A comprehensive literature search was conducted to identify relevant articles focusing on the intensive care management and specific organ-related manifestations of Toxic Epidermal Necrolysis (TEN). Electronic databases, including PubMed and the Cochrane Library, were searched from January 2000 to December 2025. The search strategy utilized a combination of Medical Subject Headings (MeSH) terms and free-text keywords, including: “toxic epidermal necrolysis,” “Stevens-Johnson syndrome,” “intensive care unit,” “critical care,” “epidemiology”, “fluid resuscitation,” “pathophysiology”, “gastrointestinal symptoms”, “wound management,” and “immunomodulatory therapy”.

The selection criteria focused on peer-reviewed articles published in English, including randomized controlled trials, observational studies, retrospective cohorts, and clinical guidelines. Review articles and case reports were also screened for cross-references to ensure a comprehensive overview. The gathered literature was narratively synthesized to present a cohesive update on current clinical practices and management controversies.

The aim of this case study is to present a TEN manifestation with multiorgan failure requiring support in the intensive care department, with special attention to a multidisciplinary approach and combined immunosuppressive therapy, and current evidence summarized in a narrative review of the literature.

## 2. Case Description

**Figure 1:**
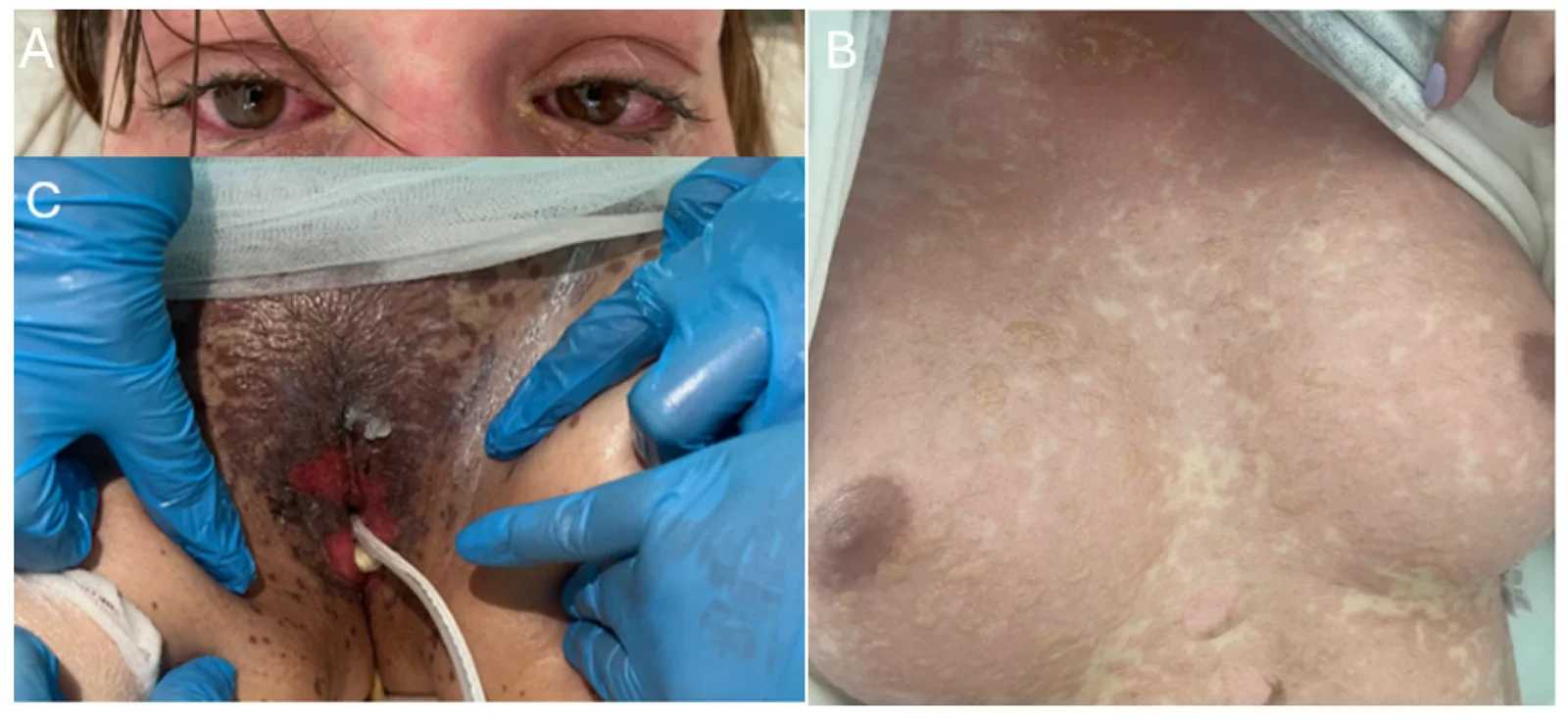
Clinical presentation of toxic epidermal necrolysis with extensive mucocutaneous involvement. (**A**) Facial erythema with conjunctival involvement. (**B**) Widespread confluent dusky erythematous lesions with blisters and epidermal detachment on the trunk and a positive Nikolsky sign. (**C**) Extensive erosive anogenital mucosal involvement.

A 25-year-old female patient was admitted on the 30 April 2025 to the Intensive Care Department due to multiorgan failure caused by TEN. The patient was first admitted to the Department of Dermatology on the 28 April 2025 with the diagnosis of SJS/TEN syndrome. Lamotrigine had been started four weeks earlier due to mood disturbances according to the standard dose-escalation regimen (25 mg once daily for the first 2 weeks, followed by 50 mg once daily for the next 2 weeks). The patient discontinued lamotrigine on her own one day before admission to the Department of Dermatology. Her chronic medications included levothyroxine, fluoxetine, and oral hormonal contraception. None of the administered drugs should cause an interaction increasing the blood concentration of lamotrigine in the blood. No other recently introduced medications were identified. Application of the ALDEN algorithm with the result of 6 points classified lamotrigine as a very probable causative agent of TEN. The ALDEN algorithm (Algorithm of Drug Causality in Epidermal Necrolysis) is a medical scoring system used to identify the specific culprit drug causing Stevens-Johnson syndrome and TEN. The criteria in the algorithm include time from drug administration to onset of symptoms, presence of the drug in the body when the symptoms began, preexposure history, whether the condition improved or worsened after stopping the drug, the frequency of the specific drug known in the literature to cause SJSJ/TEN, and finally alternative causes of the reaction. On the day of hospital admission, the patient presented with severe skin tenderness, conjunctival hyperemia, and extensive painful erosions involving the oral mucosa, tongue, buccal mucosa, and lips with hemorrhagic crusting. Dermatological examination revealed rapidly progressive, confluent dusky erythematous and atypical targetoid macules with flaccid bullae and areas of epidermal detachment involving the face, trunk, upper and lower extremities, palms, soles, and vulvovaginal mucosa. Skin involvement affected approximately 40% of the body surface area, with a positive *Nikolsky sign*. Skin biopsy was not performed due to the classic clinical presentation. The next day, due to the rapid progression of the cutaneous lesions, difficulties in controlling pain, dyspnea, and cardiovascular instability, the patient was admitted to the intensive care unit (ICU). On admission, the patient was alert and responsive, with difficulties in breathing, hypertension, tachycardia, and widespread maculopapular rash and blisters covering around 60% of the patient’s body surface area, with evident *Nikolsky sign* (Figure 1).

The patient required analgesia, intubation, and mechanical ventilation. The immunosuppressive treatment, which began in the dermatological department with one gram of intravenous Methylprednisolone administered for three days with reduction in the doses for the next days, intravenous immunoglobulins 80 g per day for three consecutive days, and etanercept (TNF alpha inhibitor) 50 mg sc once a week for three consecutive weeks, was continued. During the next days, a standard treatment including analgosedation, mechanical ventilation, bronchofiberoscopy, cardiovascular stabilization and monitoring, dexamethasone administration, fluid balance control, nutrition, rehabilitation, and microbiological surveillance was performed. The patient required several ophthalmological, laryngological, and gynecological consultations.

Local treatment consisted of non-adherent sterile dressings, emollients, and dedicated mucosal care. A topical corticosteroid ointment was applied to the anogenital area to reduce inflammation and prevent adhesions and stenosis, while oral lesions were treated with a mouth rinse containing nystatin, lignocaine, and hydrocortisone. Petroleum jelly was used for lip involvement.

During hospitalization, the patient underwent repeated bedside ophthalmological consultations. Visual acuity could not be assessed during the initial examinations due to sedation and, therefore, a lack of cooperation. In the initial phase, the assessment was limited due to the patient’s general condition, as she remained sedated and unresponsive, and the examination was performed using a handheld slit lamp. Acute ocular findings were recorded descriptively without formal Sotozono grading, as the patient’s critical condition precluded a comprehensive examination required for standardized severity assessment. At that time, the cornea remained transparent, with no fluorescein staining or only minimal epithelial involvement noted. The anterior chamber was quiet. Intensive lubrication and topical antibiotic therapy (ofloxacin administered four times daily) were recommended.

**Figure 2:**
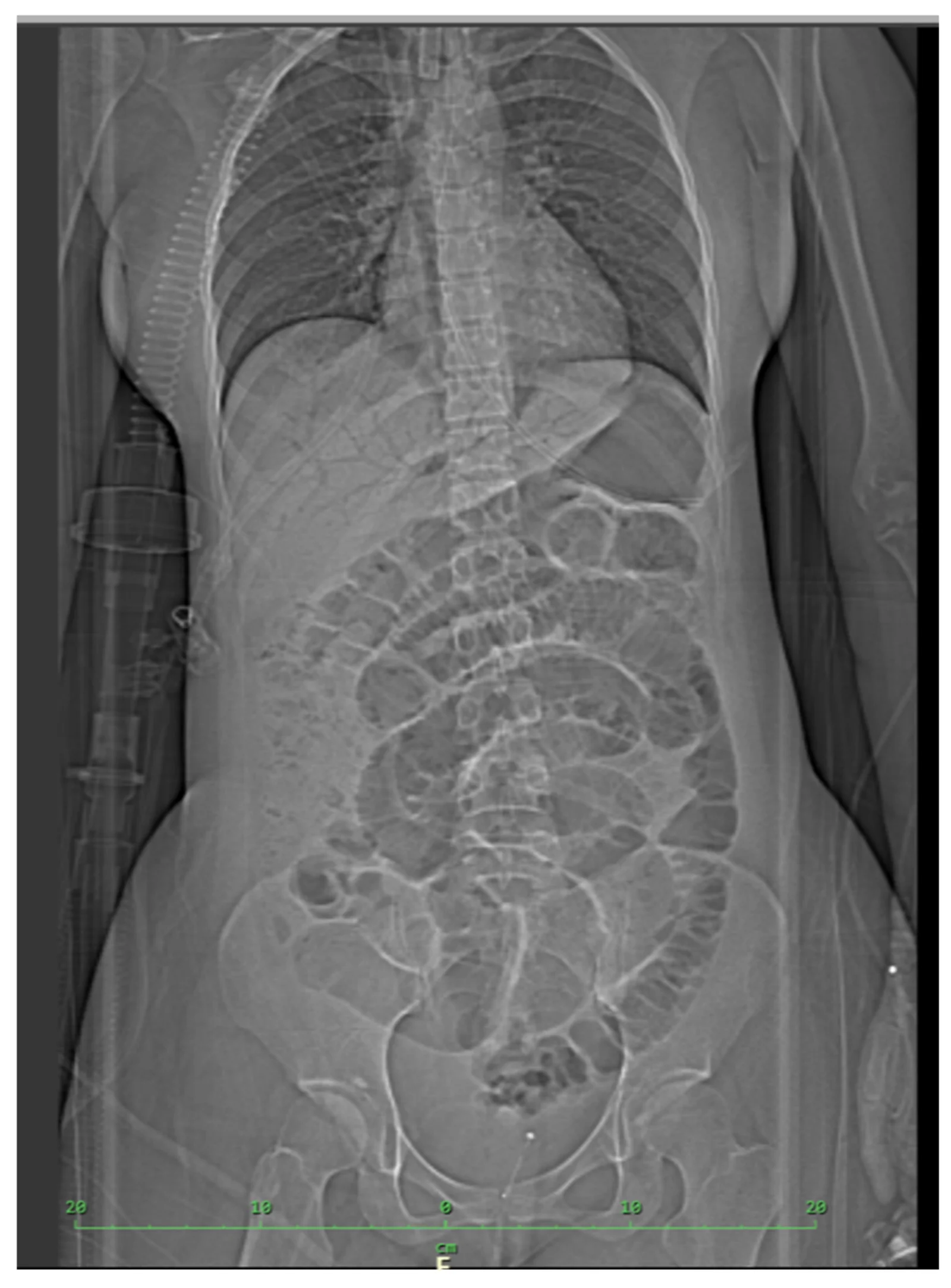
Abdominal CT imaging demonstrating extensive bowel distention in the acute phase of toxic epidermal necrolysis.

**Figure 3:**
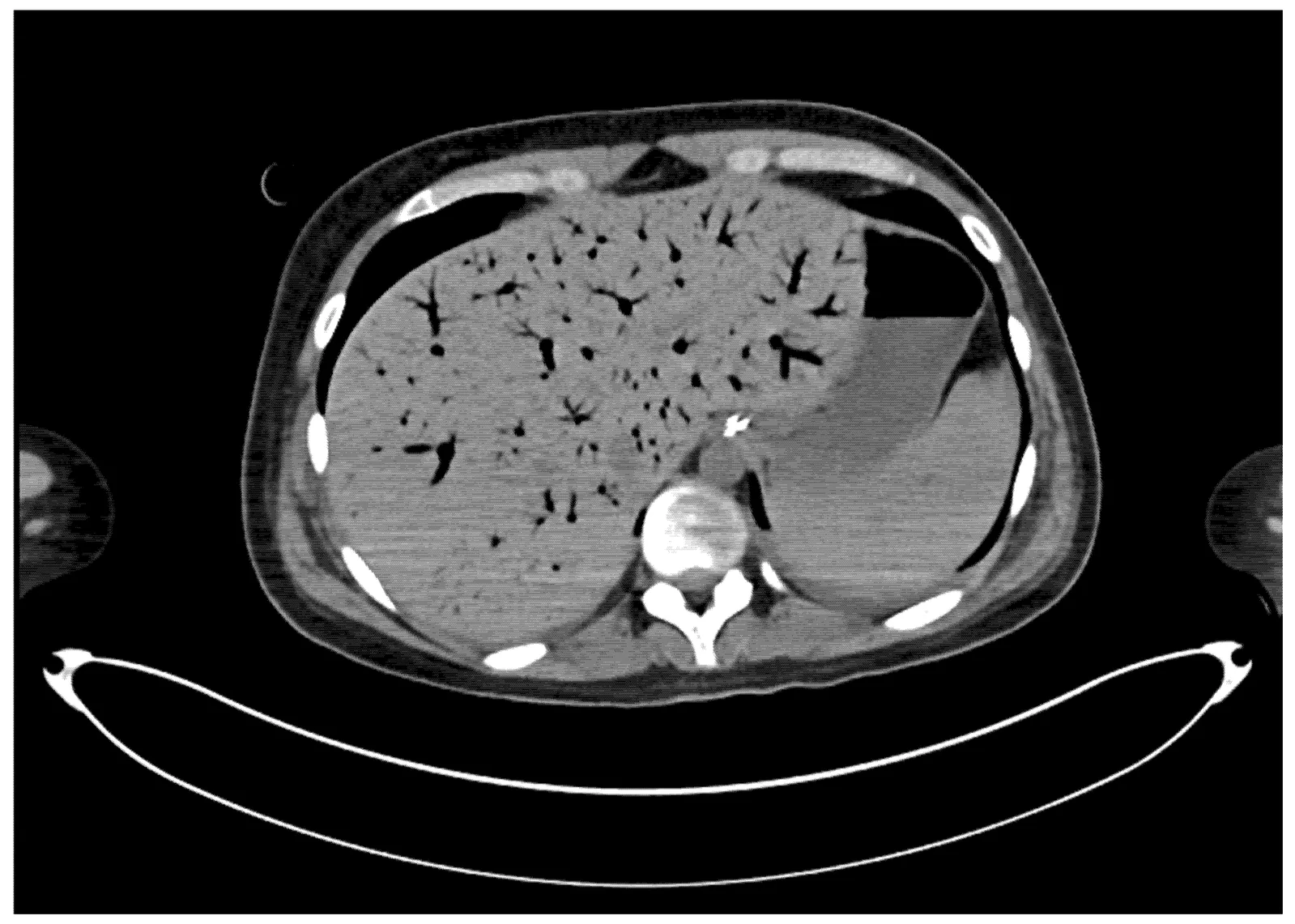
Abdominal CT imaging demonstrating extensive portal venous gas in the acute phase of toxic epidermal necrolysis.

During the ICU hospitalization, the patient suffered from a central line-related bloodstream infection and ventilator-associated pneumonia with *Klebsiella pneumoniae*, *methicillin*-*sensitive Staphylococcus aureus (MSSA*), and *methicillin-resistant Staphylococcus epidermidis (MRSE)* requiring meropenem and vancomycin treatment. On the tenth day of ICU hospitalization, a percutaneous tracheotomy under the control of bronchoscopy was performed. On the 21st day of treatment, the patient presented with vomiting and a gastric residual volume of more than 1000 mL of fecal matter collected during 6 h. An abdominal CT scan revealed the presence of fluid in the pelvis and around the liver and spleen; the intestinal loops were significantly dilated, with segmental thickening of the wall, filled with large amounts of fecal matter, with fluid levels—signs of obstruction. Due to the presence of gas bubbles in the intestinal walls, large amounts of air in the portal vein and its branches within the liver, and in the superior mesenteric vein, intestinal ischemia was suspected (Figure 2 and Figure 3).

The patient was qualified for surgery, which did not reveal any abdominal or intestinal pathology. Due to suspicion of pathological changes in the mucous membrane of the intestines related to the primary disease, plasmapheresis was commenced (five plasmapheresis procedures were performed). The patient’s general condition started to improve in the next few days. The analgosedation was decreased with the infusion of sufentanyl and ketamine for pain control. The patient started to regain consciousness, and weaning from mechanical ventilation was commenced. Dermatological examination showed marked improvement, with no new lesions, a negative *Nikolsky sign*, and only residual anogenital erythema. Systemic corticosteroids were discontinued, and topical treatment was continued.

During subsequent bedside ophthalmological evaluations, a gradual progression of ocular surface abnormalities was observed. Features of incomplete eyelid closure, epithelialization of the eyelid margins, and ocular surface dryness were noted. In later examinations, punctate epithelial defects of the cornea were described bilaterally. Early cicatricial changes also developed, including adhesions between the eyelid margins and symblepharon formation, while the cornea remained transparent.

On the 25th day of hospitalization, the patient was disconnected from the ventilator. On the 33rd day, the tracheotomy tube was exchanged for a foniatric tube. The patient started vocalization and intensive rehabilitation. Finally, the tracheotomy tube was removed. After 43 days of ICU hospitalization, the patient was discharged to the dermatological ward, where local skin treatment was continued, and the patient was discharged home on the 50th day.

**Figure 4:**
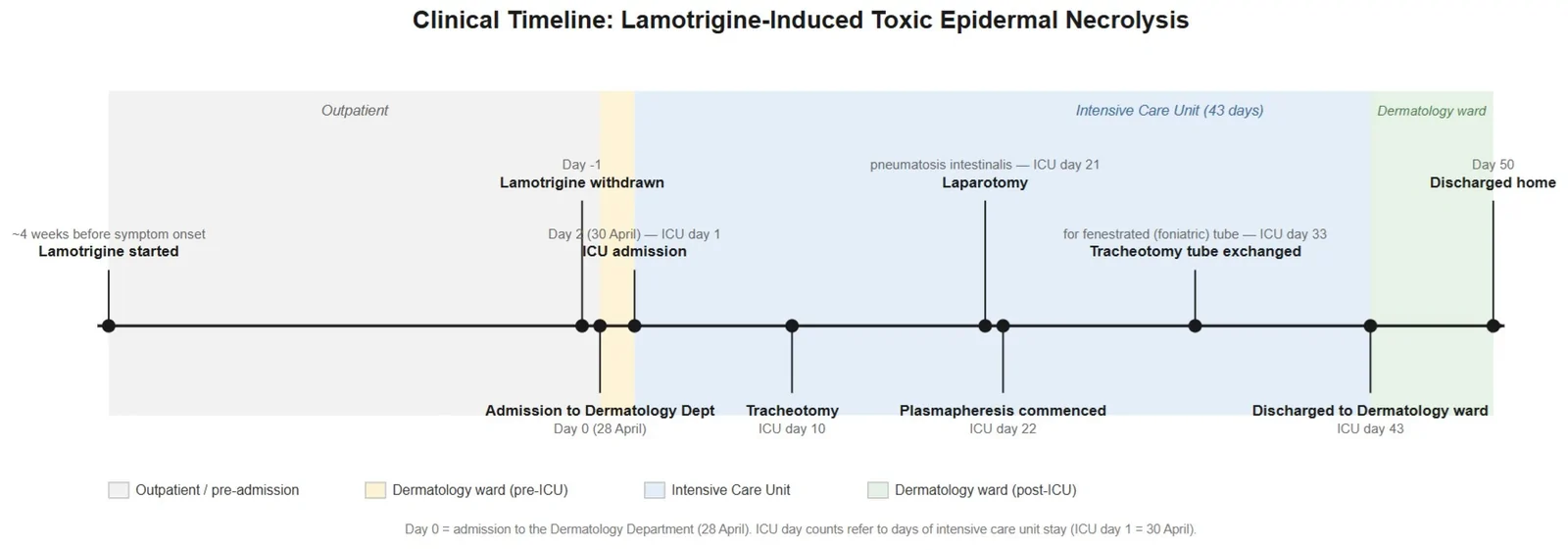
Timeline of events during the hospitalization.

Figure 4 presents a timeline of the most relevant moments during the hospitalization.

After discharge from the hospital, the patient remained under regular ophthalmological follow-up. In July 2025, she presented to the emergency department with worsening ocular symptoms, including pain, photophobia, foreign body sensation, and mucopurulent discharge. Ophthalmological examination revealed eyelid edema, meibomian gland dysfunction (MGD), conjunctival hyperemia, and mucous discharge in the conjunctival sac. The cornea remained transparent; however, diffuse punctate epithelial erosions were present. Early corneal neovascularization, predominantly from the inferior quadrants, as well as conjunctival adhesion in the medial canthus, were also noted.

During subsequent outpatient visits, gradual improvement was observed, with a reduction in pain and photophobia. However, features of ocular surface disease persisted, including MGD, conjunctival hyperemia, and punctate epithelial erosions. Tear film instability was also present. Trichiasis was identified with misdirected eyelashes contacting the corneal surface. Mechanical removal of aberrant eyelashes was performed. Topical treatment was continued and modified, including corticosteroids, preservative-free lubricants, eyelid hygiene, and topical ciclosporin.

At the most recent outpatient visit, best-corrected visual acuity was 0.7 in the right eye and 0.5 in the left eye, showing partial improvement compared to the early post-discharge period and subsequent stabilization during follow-up. The patient reported subjective improvement, although symptoms fluctuated, with periods of increased ocular discomfort, dryness, and occasional burning sensation. On examination, features of ocular surface disease were still present, including signs of MGD and mild conjunctival hyperemia. The patient continues topical treatment with ciclosporin eye drops once daily, topical corticosteroid twice daily, and frequent use of preservative-free lubricating drops and gel formulations. Eyelid hygiene with warm compresses was also recommended.

Given the clinical course and known risk of chronic ocular complications in SJS/TEN, the patient remains under regular ophthalmological supervision.

## 3. Discussion

The management of SJS/TEN is multifaceted and should include a prompt diagnosis and identification of the trigger, followed by its cessation and subsequent supportive care in a specialist burns ICU or similar high-dependency unit. Due to the severity of the SJS/TEN, the UK guidelines advise that all patients with skin changes covering > 10% of BSA should be managed in an ICU/burn center with complex nursing care and physicians experienced in its diagnosis and management [17]. It is advisable to transfer those patients to a specialized center within 7 days to improve survival [18].

*Diagnosis and Prognostication*

Recognition of TEN relies on identifying the combination of acute onset, extensive epidermal detachment, severe mucosal involvement, and recent drug exposure. In the presented case report, the patient presented with mucocutaneous symptoms of SJS/TEN about 4 weeks after exposure to the causative agent. The ALDEN algorithm classified lamotrigine as a very probable causative agent of the disease. In selected cases, skin biopsy and additional diagnostic testing may be warranted to support the diagnosis and to distinguish TEN from other blistering disorders, helping to guide appropriate management and improve outcomes. A thorough history is important to identify the causative agent. The symptoms of the disease typically appear within 8 weeks of exposure to the responsible drug, with most cases presenting between 4 days and 4 weeks after starting the therapy [1]. The clinical presentation is not always straightforward and may resemble other blistering disorders. There are some studies investigating the potential role of biomarkers, such as granulysin and the CCL-27 cytokine, in the diagnosis of SJS/TEN, in addition to their role in the disease’s pathogenesis. A study performed by Abe et al. showed that granulysin levels were elevated in the serum of 4 out of 5 patients, even before cutaneous and mucosal involvement, while there were no elevations in the control group [19]. CCL-27 is a nonspecific cytokine whose levels are elevated in the serum of SJS/TEN patients and which correlate with disease activity. CCL-27 is highly expressed in the skin lesions of SJS/TEN patients and is associated with the trafficking of T cells to the site of inflammation in the skin. It is believed that CCL-27 is produced by keratinocytes in the skin lesions of SJS/TEN patients and then released into circulation [20]. Despite the increased levels of both granulysin and CC27 in the serum of patients with SJS/TEN, both these markers are non-specific, and their clinical utility in the early diagnosis is limited [21]. Although HLA genotyping is recommended before initiating selected high-risk medications in certain Asian populations, routine HLA testing is not currently recommended in European clinical practice for patients with suspected lamotrigine-induced SJS/TEN and was therefore not performed in our patient.

The most widely used prognostication scale is the severity of illness score for the TEN scale (SCORTEN), which was verified as a reliable predictive score [22,23]. The SCORTEN score of the patient presented in this case scenario on admission to the ICU was 3 points, with a mortality risk of 35%, due to tachycardia, elevated urea concentration, and BSA involvement of more than 10%. The favorable outcome of the presented patient might be due to early diagnosis and prompt multidisciplinary treatment. It should be noted that due to advances in the management of TEN, it is possible that the SCORTEN overestimates mortality, especially in centers with experience in treating those patients [24]. There is also a case series of 10 patients with SJS/TEN treated with a single dose of etanercept (50 mg subcutaneous injection) with all patients surviving despite an SCORTEN-predicted mortality of 50% [25]. Two separate meta-analyses found a significant reduction in mortality risk with cyclosporine treatment compared with SCORTEN-predicted mortality [26,27].

An alternative prognostic scale is the ABCD-10 algorithm, which accounts for prior dialysis and is also considered a reliable predictor of SJS/TEN [28]. Both scoring systems are presented in Table 1. One of the most challenging elements in evaluating the severity of SJS/TEN is estimating the BSA affected by the disease. According to Creamer et al., only epidermis that is already detached and epidermis that is detachable (positive *Nikolsky sign*) should be included in the calculation of the BSA affected by the disease [17].

*Supportive and Intensive Care Management*

Patients with SJS/TEN treated in the intensive care unit should be managed according to general standards, with supportive care being the mainstay of treatment, which includes cessation of the responsible drug, cardiovascular stability with special attention to fluid and electrolyte management, infection control, and wound care [29,30]. The presented case report of a patient requiring prolonged hospitalization in the intensive care unit confirms the complexity of management of SJS/TEN patients with multiorgan failure.

Furthermore, it is recommended to include routine dermatology, ophthalmology, and gynecology consultation, as skin, eye, and vaginal involvement can be overlooked, causing lifelong morbidity [13,31,32]. Mechanical ventilation and intubation in order to protect the airway should be considered in patients with significant oropharyngeal and airway involvement [33,34]. There are proposed indications for intubation and tracheostomy in patients with SJS/TEN. If patients experience oral involvement plus one of the following: BSA > 70% on admission; a >15% increase in BSA from day 1 to day 3; an underlying neurological diagnosis that may impair airway protection; or airway involvement on direct laryngoscopy, intubation should be commenced [30]. Oral care with regular irrigation using an antiseptic solution or plain mouthwash with viscous lidocaine should be a standard management. Frequent application of a lubricant, such as Vaseline, to the lips is also recommended [13]. Fluid and nutrition should be monitored, especially in patients with oral mucosa involvement, which may prevent normal oral intake and when cutaneous lesions increase insensible losses [13].

Fluid requirements seem to be of similar importance as in burn patients; however, the amount of fluids administered appears to be 30% lower than in burn patients with similar BSA affected [35]. Regarding fluid therapy, especially with the infusion of large amounts of fluids, it is important to remember that administered fluids can affect acid-base status [36]. Enteral feeding should be commenced as soon as possible according to the ESPEN guidelines [37]. Early enteral nutrition has been proven to be beneficial in treating patients with serious, complicated wounds [18]. A review from the US, conducted in patients at burn centers, showed that most required supplemental nutrition because they were unable to meet their nutritional targets orally [38]. A case series including 40 patients with SJS/TEN showed that 65% of patients required nasogastric enteral feeding [39].

*Gastrointestinal Manifestations*

Gastrointestinal symptoms and complications are observed in about 10% of patients with SJS or TEN and are usually mild [40]. However, the intestinal involvement worsens the prognosis of these patients, with fatal outcome reaching even 44% of cases [41]. In our case report, the patient also developed severe abdominal symptoms with vomiting, abdominal distention, and increased intra-abdominal pressure. The CT scan revealed symptoms of pneumatosis intestinalis and air in the portal vein, with no pathological findings during the laparotomy performed due to suspicion of intestinal perforation or bowel ischemia.

Gastrointestinal involvement in TEN is thought to result from the same immunopathogenic mechanisms that cause epidermal necrolysis, including massive keratinocyte apoptosis mediated by cytotoxic T cells and soluble mediators such as Fas ligand and granulysin [41]. Gastrointestinal symptoms usually appear within two weeks of cutaneous lesions, but can even develop many weeks after the first cutaneous symptoms. Most commonly, gastrointestinal bleeding, diarrhea, abdominal pain, abdominal distension and dysphagia can be observed [41]. Rarely, life-threatening mesenteric ischemia, intra-abdominal abscess, intestinal infarction and intestinal obstruction can be diagnosed in SJS/TEN patients [41,42]. There are also various endoscopic findings observed in SJS/TEN patients, including hyperemia, friability, erosions, superficial or deep ulcerations, pseudomembrane formation, necrotic plaque formation with mucosal sloughing [41]. Liver involvement with asymptomatic hepatic enzyme elevation, hepatitis, cholestatic hepatitis and hepatic failure can all also affect SJS/TEN patients [40,43].

In one reported case, a patient with TEN developed abdominal distension, and imaging revealed pneumatosis intestinalis and portal venous gas; subsequent surgical findings showed patchy nonperforated necrosis of the small bowel, supporting a direct association between TEN and pneumatosis intestinalis via mechanisms of epithelial apoptosis and ischemic injury [42]. Intestinal pneumatosis is a rare condition defined as pathological gas infiltration into the bowel wall. Intestinal pneumatosis can be observed in many clinical conditions, including necrotizing enterocolitis (NEC) in infants, intestinal infarction, inflammatory bowel disease, connective tissue disease, surgery, immune deficiency, or chemotherapy [44]. There is a primary intestinal pneumatosis contributing to 15% of all cases, which is an idiopathic gas infiltration of the bowel wall termed Pneumatosis Cystoides Intestinalis. The more frequent secondary form contributes to 85% of cases and can be due to local bowel pathology (ischemia, obstruction, cancer, iatrogenic) or systemic pathology (immunodeficiency, drugs, transplantation, autoimmune disease).

The pneumatosis intestinalis is thought to involve extensive epithelial cell death and disruption of the mucosal barrier, increased mucosal permeability, which can allow intraluminal gas to dissect into the bowel wall, eventually spreading in mesenteric and portal vessels—portomesenteric vein gas (PMVG) [44]. Pneumatosis intestinalis related to bowel ischemia and infarction is a life-threatening condition requiring prompt diagnosis and surgical management. Conversely, pneumatosis intestinalis due to non-ischemic conditions commonly shows a less aggressive clinical course and frequently requires conservative management rather than the surgical approach [44]. Interestingly, in the SJS/TEN case report, the patient presented with symptoms of acute abdominal disorder, with suspicion of intestinal ischemia due to pneumatosis intestinalis. However, as mentioned, the laparotomy did not show any ischemic pathology on inspection, as the symptoms were probably caused by epithelial cell death, disruption of the mucosal barrier, and increased mucosal permeability.

*Infectious Complications and Wound Care*

Currently, there is no consensus regarding the optimal and standardized management of wounds in SJS/TEN patients. In the presented case, a conservative approach was advocated with antishear measures and a nonadherent sterile dressing, leaving the detached skin in place.

In SJS/TEN, epidermal detachment occurs at the epidermal-dermal junction, but the dermis and its collagen and reticular fibers are preserved [45]. Due to dermis preservation, there is less scarring after healing in comparison to second-degree burns [46]. Protection of the dermis from infection, shearing, and injury during healing is of utmost importance to minimize scarring. Careful dressing changes and sufficient moisture levels are essential for maintaining healthy dermis and preventing scarring, infection, heat loss, and dehydration [47].

Some centers favor a surgical approach, which involves debriding the detached epidermis and applying biosynthetic dressings, allografts, or xenografts for physiologic wound closure.

A study performed by McCullough et al. described a series of SJS/TEN patients who were managed with surgical wound debridement and subsequent coverage with antimicrobial dressing. The authors showed a 10% mortality rate, lower than 16% predicted by the SCORTEN scale; the reduction in mortality couldn’t be attributed to surgical wound management, as the authors included some other therapeutic interventions, like the administration of IVIg and steroid cessation [39]. Removal of the denuded epidermis, which can serve as a nidus for infection, can limit cutaneous infections that impair reepithelialization or lead to sepsis. According to this surgical approach, debridement is essential for achieving a clean wound bed before healing, preventing wound infection, and providing a clean surface for biological dressing application. Wound debridement also potentially leads to faster wound healing than conservative management [6,29,39]. Given the fact that cytokines in blister fluid may recruit cytotoxic T cells to the epidermis and lead to further apoptosis, early removal of the blistered epidermis and blister fluid may help limit disease progression [46]. Debridement of blisters may be considered an approach reserved for patients with greater than 30% BSA of epidermal detachment, those who have failed conservative management, or if the disease is not well controlled and blistering continues to spread.

With the conservative approach, the detached epidermis is left in situ, and it acts as a skin graft or biologic dressing to facilitate wound healing and protect the dermal layer underneath [10,13]. This approach aids in preserving the viable dermis, allowing for reepithelialization. A study published by Dorafshar et al. also showed a reduction in mortality with the anti-shear therapy, in which blister fluid was aspirated, and the denuded epidermis was left in place to act as a biological skin graft. Due to beneficial results, the institution made a transition from debridement to antishear. The authors concluded that this type of therapy could be an alternative to surgical debridement [48]. In comparison to burned patients, the damage in SJS/TEN occurs between viable layers of skin, and some epidermal cells are detached but not directly injured by the inflammatory process. In that case, layers of skin may still retain some biological function, so epidermal remnants may have some benefit if left in situ. The skin should be covered with topical emollients and nonadherent dressings that help to maintain barrier function, limit fluid and heat loss, aid reepithelialization due to fewer dressing changes and ease of application [49]. The conservative approach also reduces the costs and pain and discomfort for patients associated with operative debridement and dressing changes. The main problem with this approach is the risk of bacterial colonization with *Staphylococcus aureus* and *Pseudomonas aeruginosa*.

Management of infection is another important element in the management of patients with SJS/TEN; prophylactic or early empiric antibiotics do not improve outcome [18,50]. Patients with BSA involvement > 30% were at higher risk of developing bacteremia, which was associated with increased risk of in-hospital mortality [45]. De Prost et al. in a single-center study showed that the median time to a bloodstream infection was 11 days from the start of the symptoms and five days from the beginning of the hospitalization [51].

*Systemic Immunomodulatory Treatment*

Given the immune-mediated pathogenesis of TEN, several systemic immunomodulatory therapies have been proposed, including corticosteroids, intravenous IVIg, cyclosporine, and TNF-α inhibitors [52,53,54,55,56]. However, because of the rarity of the disease, the available evidence is limited, and no universally accepted evidence-based standard immunomodulatory treatment has been established [21]. In our patient, treatment decisions were guided by the rapidly progressive clinical course, with extensive epidermal detachment, multiorgan failure, and subsequent gastrointestinal complications. Consequently, a multimodal immunomodulatory approach combining corticosteroids, IVIg, etanercept, and later plasmapheresis was adopted. This strategy was based on the available evidence and clinical judgment, recognizing that no standardized evidence-based treatment algorithm for TEN currently exists.

There is still a debate about the beneficial role of systemic steroids in the treatment of SJS/TEN patients. High-dose steroids early in the course of disease are prone to diminish inflammation, with decreasing concentration of Il-6 [57]. On the other hand, there are concerns with the use of steroids, which might increase the risk of complications like prolonged wound healing, risk of infection, occult early signs of sepsis, gastrointestinal bleeding, and increased mortality. A large retrospective study of 281 patients found a trend for beneficial effect of corticosteroids over the patients treated with standard supportive care [58]. Furthermore, another study on 442 patients with TEN and SJS found an improved survival rate in patients treated with steroids versus standard supportive care alone [7]. A meta-analysis performed by Zimmermann et al., including 11 studies comparing the use of steroids versus supportive treatment, found a positive effect (although statistically insignificant) related to the use of steroids [59].

According to results analyzing IVIg used as monotherapy, there are no mortality benefits associated with these regimens [4,17,20,59,60,61,62]. Potential mechanism of action of IVIg is the blockade of the FAS receptor and inhibition of Fas-mediated keratinocyte death [63], a decrease in NK cells in the peripheral blood, and decreased release of granzyme B in plasma [64]. A study including 23 patients with TEN treated with IVIg versus supportive care alone showed no benefits [65]. Another larger retrospective study analyzing 64 patients also showed no mortality benefit regardless of IVIg dose [17]. The researchers point towards biased results of these studies, as there are some retrospective data showing that physicians are more likely to administer IVIg in more severe cases, with higher BSA involvement, which, of course, are predisposed to a worse outcome [10,13].

However, there are some beneficial studies on the combined use of steroids and IVIg [10,66,67,68]. A meta-analysis including 26 studies proved that combination therapy reduced the recovery time and the length of hospitalization, whereas its impact on reducing mortality was not significant [66]. A retrospective comparative study in 20 patients receiving combination therapy with steroids and IVIg showed a tendency to reduced mortality with combination therapy compared to steroids alone (no statistical significance). Combination therapy also arrested disease progression earlier and decreased hospitalization time [67]. Another study including 24 patients receiving combination therapy of IVIg and steroids also showed a reduced duration of hospitalization and a trend toward reduced mortality with this approach [68].

Cyclosporine is a less studied drug administered in SJS/TEN in comparison to steroids. However, its suppressive action on activated T lymphocytes might play an important role in the treatment of SJS/TEN by inhibiting cytotoxic T-cell-derived granulysin production responsible for keratinocyte apoptosis [1]. There have been some small case series or case reports that had favorable outcomes related to cyclosporine administration [1]. There are also results of two meta-analyses showing positive effects related to the use of regimens including cyclosporine, both showing a significant survival benefit [27,69].

An observational study by Han et al. showed that patients receiving plasmapheresis in the course of treatment for SJS/TEN had lower severity of illness scores, which include mucosal lesions, cutaneous lesions, and general condition during the next days of the disease [70].

Given that hemadsorption technology shares the mechanistic principle of removing circulating inflammatory mediators and toxins, the reported benefits of plasmapheresis and other blood purification modalities in TEN provide a theoretical rationale for future investigation of hemadsorption as a treatment option. The current literature confirms that hemadsorption is technically feasible, safe in the short term, and capable of removing target molecules in other critical illnesses, but emphasizes that it remains experimental and unproven in TEN [71,72].

There are also studies showing some beneficial effects of antitumor necrosis factor agents in the treatment of SJS/TEN. An analysis by Zhang et al. reported positive outcomes in almost 90% of patients with the use of TNF-α inhibitors [73]. An RCT showed an 8.3% mortality rate, which was lower than predicted by SCORTEN (17.7%) and lower than the mortality associated with steroid use (16.3%); however, neither result was statistically significant [74]. There is a case series of 10 patients with SJS/TEN treated with a single dose of etanercept, with all patients surviving despite SCORTEN predicted mortality of 50% [25]. It is, however, important to remember that despite the effectiveness of TNF -alpha antagonists in halting the progression of TEN/SJ, they are potent immunosuppressants and the risk of infection must be considered in each case.

*Ocular Complications and Long-Term Outcomes*

Ocular involvement is a frequent component of SJS/TEN and may occur in both the acute and chronic phases of the disease. In the acute stage, patients often present with conjunctival hyperemia, photophobia, and epithelial defects, which in more severe cases may progress to pseudomembrane formation and corneal involvement [21,75]. The intensity of ocular surface inflammation at this stage is considered one of the key factors influencing long-term visual outcomes.

In the chronic phase, patients may develop persistent ocular surface disease, most commonly presenting as severe dry eye, MGD, and conjunctival scarring [75]. In more advanced cases, this may lead to symblepharon formation, limbal stem cell deficiency, and progressive visual deterioration, especially in patients without early ophthalmological management [21]. Therefore, regular ophthalmologic follow-up and early initiation of appropriate topical therapy are crucial to limit long-term complications. A limitation of this report is the absence of formal acute-phase Sotozono ocular severity scoring. During the initial ophthalmological consultations, the patient remained sedated, mechanically ventilated, and critically ill in the intensive care unit, making a comprehensive slit-lamp examination and formal severity grading impractical. Consequently, acute ocular findings were documented descriptively rather than according to the Sotozono classification. This limits direct comparison with published cohorts in which standardized Sotozono scores have been shown to correlate with long-term ocular outcomes.

## 4. Conclusions

SJS and TEN are rare, severe, and life-threatening dermatological conditions that require rapid recognition and immediate elimination of the culprit medication. Early diagnosis is crucial, as disease progression can be rapid and associated with high morbidity and mortality. The cornerstone of management is treatment in a specialized facility capable of providing high-level supportive care, supported by a multidisciplinary team involving dermatologists, intensivists, ophthalmologists, gynecologists, and other specialists as needed. Systemic therapy may play an important adjunctive role; however, due to the rarity of SJS and TEN and the limited availability of high-quality evidence, the optimal immunomodulatory treatment strategy remains uncertain.

## Figures and Tables

**Table 1:** SCORTEN (Severity-of-Illness Score for Toxic Epidermal Necrolysis) and ABCD-10 scales for SJS/TEN severity evaluation.

Scorten		ABCD-10	
Parameter	Weight	Parameter	Weight
Age ≥ 40 years	1	Age ≥ 50 years	1
Malignancy—Yes	1	Serum bicarbonate <20 mmol/L	1
BSA detached > 10%	1		
Serum bicarbonate <20 mmol/L	1	Active cancer—Yes	2
Serum urea nitrogen >28 mg/dL	1	Dialysis prior to admission—Yes	3
Serum glucose >252 mg/dL	1	BSA Involvement > 10%	1
Tachycardia ≥ 120 bpm	1		
Maximum score possible	7		8

BSA—body surface area; SJS—Stevens-Johnson Syndrome, TEN—Toxic Epidermal Necrolysis.

## Data Availability

The original contributions presented in this study are included in the article. Further inquiries can be directed to the corresponding author.

## Abbreviations

The following abbreviations are used in this manuscript:

TEN	Toxic Epidermal Necrolysis
SJS	Stevens–Johnson syndrome
HLA	Human Leukocyte Antigen
BSA	Body Surface Area
ICU	Intensive Care Unit
MSSA	Methicillin-sensitive Staphylococcus aureus
MRSE	Methicillin-resistant Staphylococcus epidermidis
TNF	Tumour Necrosis Factor
MGD	Meibomian gland dysfunction
